# Open science and Big Data in South Africa

**DOI:** 10.3389/frma.2022.982435

**Published:** 2022-11-07

**Authors:** Tony Hey

**Affiliations:** Scientific Computing Department, Rutherford Appleton Laboratory, Science and Technology Facilities Council/UK Research and Innovation (STFC/UKRI), Didcot, United Kingdom

**Keywords:** Open Science, SKA project, FAIR data, neutron data, research data management (RDM)

## Abstract

With the Square Kilometer Array (SKA) project and the new Multi-Purpose Reactor (MPR) soon coming on-line, South Africa and other collaborating countries in Africa will need to make the management, analysis, publication, and curation of “Big Scientific Data” a priority. In addition, the recent draft Open Science policy from the South African Department of Science and Innovation (DSI) requires both Open Access to scholarly publications and research outputs, and an Open Data policy that facilitates equal opportunity of access to research data. The policy also endorses the deposit, discovery and dissemination of data and metadata in a manner consistent with the FAIR principles – making data Findable, Accessible, Interoperable and Re-usable (FAIR). The challenge to achieve Open Science in Africa starts with open access for research publications and the provision of persistent links to the supporting data. With the deluge of research data expected from the new experimental facilities in South Africa, the problem of how to make such data FAIR takes center stage. One promising approach to make such scientific datasets more “Findable” and “Interoperable” is to rely on the Dataset representation of the Schema.org vocabulary which has been endorsed by all the major search engines. The approach adds some semantic markup to Web pages and makes scientific datasets more “Findable” by search engines. This paper does not address all aspects of the Open Science agenda but instead is focused on the management and analysis challenges of the “Big Scientific Data” that will be produced by the SKA project. The paper summarizes the role of the SKA Regional Centers (SRCs) and then discusses the goal of ensuring reproducibility for the SKA data products. Experiments at the new MPR neutron source will also have to conform to the DSI's Open Science policy. The Open Science and FAIR data practices used at the ISIS Neutron source at the Rutherford Appleton Laboratory in the UK are then briefly described. The paper concludes with some remarks about the important role of interdisciplinary teams of research software engineers, data engineers and research librarians in research data management.

## Big Scientific Data comes to South Africa

With the Square Kilometer Array (SKA) project (The SKA Project, 2022) and the new Multi-Purpose Reactor (MPR) to replace the existing Safari-1 neutron source (Necsa, 2022) soon coming on-line, South Africa and the other collaborating countries in Africa will have to cope with an increasing deluge of scientific data. In a talk given in January 2007, Turing award winner Jim Gray outlined the existing three paradigms of scientific discovery: first, empirical observations; second, theoretical explorations; and third, computational simulations. He then identified the emergence of a fourth paradigm: data-intensive scientific discovery (Hey et al., 2009):

“*The techniques and technologies for data-intensive science are so different that it is worth distinguishing data-intensive science from computational science as a new, fourth paradigm for scientific exploration.”*

Gray's fourth paradigm builds on the first three paradigms of observation, theory, and computation and requires that research scientists develop new skills in data management and data analysis. The breakthrough in image classification and recognition with “Deep Learning” artificial neural networks in 2012 (Sejnowski, 2018) has already transformed much of the commercial world and is now beginning to have a major impact on scientific data analysis (Stevens et al., 2020). The management, analysis, publication, and curation of “Big Scientific Data” will soon be an important component of open science in South Africa.

This paper is focused on data aspects of the Open Science agenda and on the management and analysis of scientific data sets. Borgman's book on “Big Data, Little Data, No Data: Scholarship in the Networked World” provides an excellent introduction to scientific data policy and practice, as well as discussing some case studies in data scholarship (Borgman, 2015). For the sciences, Borgman uses the example of astronomy as a “Big Data” research field and the example of sensor-networked science as a “little data” research field. However, the extreme data rates and volume of data from the SKA project will be at a totally different scale than any previous astronomy project. The SKA project therefore has the potential to be truly transformative for science and technology in Africa. South Africa will be the location for one of the SKA project's two Science Data Processors (SDP) and for an African SKA Regional Center (SRC). The output of the SDP will be distributed to a global network of SRCs which will produce science-ready data products and provide users with the necessary software tools for analyzing the data.

After a description of the data challenges and opportunities that will be posed to African scientists by SKA-Mid, the South African component of the SKA, the implications of the draft South African National Open Science policy (Pienaar, 2022) are discussed. For research funders globally, there is now an increasing focus on Scientific Data Management plans and the linking of the full text open access papers to the relevant supporting research data. Some evidence for better compliance by researchers with this component of Open Science is provided by the improving situation in the US and the UK.

The draft South African Open Science policy requires not only open access to the full text of the research paper but also access to the digital data necessary to validate the research findings described in the paper, as well as the availability of the software that was used to analyse the data. In addition, the draft policy also specifically requires that the research data should be “FAIR” compliant (FAIR, 2022). The FAIR initiative emphasizes the importance of having machine actionable metadata for interoperability rather than just community-agreed file formats. One approach to implementing FAIR data that has been adopted by the biosciences community is to extend the standard, industry-supported Schema.org vocabulary with specific types relevant to scientific datasets.

Another source of significant scientific datasets in South Africa – though not on the scale of SKA – will be from the beamlines to be built at the new MPR reactor facility. This will be a replacement for the Safari-1 reactor which is one of the top four medical radioisotope producers in the world as well as supporting a wide range of research and applications using their Neutron Diffraction Facility. The MPR project is now in the design phase with construction planned to start in 2025. A new Neutron Beam Line Center is being planned and a new software stack comprising the instrument control system, user interface and data reduction and analysis components will need to be built (Marais, 2022). In addition, the data management processes will need to support the generation of FAIR data. A short section describing the research data management practices and the progress toward generating FAIR datasets at the ISIS Neutron source at the UK's Rutherford Appleton Laboratory is included to illustrate the important components of such a FAIR data pipeline.

The paper ends with some remarks about the need for interdisciplinary teams in research data management and the important roles of research software engineers, data scientists and research librarians.

## The SKA project in Africa

The Square Kilometer Array (SKA) project is an international effort to build the world's largest radio telescope, eventually covering over a square kilometer of collecting area. The data generated by the SKA has the potential to answer many open questions in modern astrophysics, ranging from mapping the early cosmic history of the universe to understanding how galaxies form and evolve (Scaife, in press). The most suitable telescope locations for the SKA were determined to be in remote Western Australia, around 800 km north of Perth, and in the Karoo region in the Northern Cape of South Africa, where the national government has created a radio-quiet astronomy reserve.

The first of the two SKA telescopes will operate at low radio frequencies where radio signals have wavelengths of several meters. In this first phase of the SKA, the SKA1-LOW telescope comprising 130,000 dipole antennas will be sited in Western Australia. The second of the SKA telescopes will operate in the mid-frequency radio band where radio signals have wavelengths from around one meter to tens of centimeters. The desert regions of South Africa provide the perfect radio quiet location for this mid-frequency array, SKA1-MID (SKA South Africa, 2022). The technology for the SKA1-MID instrument will use familiar-looking radio dishes to receive incoming radio signals and, when complete, will comprise 197 individual radio dishes separated by distances of up to 200 km. The second phase of the SKA project (SKA2) will extend the mid-frequency dish array into the eight other SKA African partner countries – Botswana, Ghana, Kenya, Madagascar, Mauritius, Mozambique, Namibia, and Zambia.

In his talk in 2007 in which Jim Gray identified the emergence of a fourth paradigm of data-intensive scientific exploration and discovery, he went on to say (Hey et al., 2009):

“*People now do not actually look through telescopes. Instead, they are ‘looking' through large-scale, complex instruments which relay data to datacenters, and only then do they look at the information on computers. The world of science has changed, and there is no question about this. The new model is for the data to be captured by instruments or generated by simulations before being processed by software and for the resulting information or knowledge to be stored in computers. Scientists only get to look at their data fairly late in this pipeline.”*

For the first generation of large-scale experimental facilities, it was possible to conceive of building a single facility that was able to provide end-to-end coverage of the data processing, storage and archiving needs of its users. However, with the new generation of large-scale projects, such as the Large Hadron Collider (LHC) at CERN and the global SKA Observatory project, this approach is no longer feasible. For example, the Worldwide LHC Computing Grid (WLCG) is an international collaborative project that consists of a grid-based computer network infrastructure incorporating over 170 computing centers in 42 countries (Worldwide LHC Computing Grid, 2022). It was designed by CERN to handle the many Petabytes of data produced by Large Hadron Collider (LHC) experiments. In similar fashion, the global SKA Observatory (SKAO) will require building a network of SKA Regional Centers (SRCs) to receive data from the two SKA Science Data Processors (SDPs).

When the two SKA telescopes become fully operational in the mid to late 2020s, the output data products are estimated to amount to approximately 300 Petabytes per telescope per year. Such “Extreme Scientific Data” will require the creation of a novel Science Data Processor (SDP) at each telescope that will be a schedulable part of the telescope (Chrysostomou, 2019). The goal is to reduce the raw data volume at the SDP before delivery to users. However, as for the CERN LHC data, there will also be a need to create a global network of SKA Regional Centers (SRCs) with one located in South Africa. Such a network is needed because the data volumes are so large that direct delivery to a distributed global community of end users is unfeasible. Moreover, the SKA data, as delivered to the SRCs from the telescope *via* the SDP, needs further processing to be in a state suitable for scientific analysis and publication.

Discussions about the precise roles of the SRCs are still ongoing but the SKAO has always had a very strong commitment toward implementing Open Science (Garrido et al., 2021):

“*The SKAO and the SRC network are working to enable best practices that make data and other digital research objects (e.g., algorithms, tools, workflows, protocols, or services) ‘findable, accessible, interoperable, and reusable' (FAIR). In particular, the SRC Coordination Group defined different requirements related to open science, highlighting the requirement of ‘open access,' which relates to the need for public links to SKA science data products, and the ‘reproducibility: provenance and workflow preservation' requirement, meaning that the SRCs must be capable of saving the provenance and workflow associated with the data products generated at each SRC.”*

Remarkably, the SKAO is believed to be the first large-scale facility to include reproducibility as one of the scientific metrics of its success^1^.

The South African government sees the SKA project as a catalyst for bringing new technology and skills to the African continent (Ratcliffe, 2022):

“*Aside from the benefits to African science, Big Data capabilities could be our biggest spin-off from the SKA project. The innovations, skills development and commercial potential emerging as a result of the project are huge. The potential is not just academic – we develop the taxpayer-funded intellectual property to a point where it's ready to become commercialized and benefit the economy. We will increasingly be an incubator of science and technology innovation.”*

These new skills will involve exploitation of appropriate AI and Deep Learning technologies in both the SKA data pipeline and the analysis of the SKA data products (SKA South Africa, 2022).

## Open access and Open Science

The South African Draft National Open Science Policy (Pienaar, 2022) defines Open Access as:

“*a set of principles and a range of practices through which research outputs are distributed online, free of cost or other access barriers”*.

Open Science is defined as:

“*research and development that is collaborative, transparent and reproducible and whose outputs are publicly available”*.

The policy will be applicable to all publicly funded research outputs and will require access to infrastructure at an institutional and national scale that supports the deposit, discovery and dissemination of data and metadata. One of the key guidelines for the successful implementation of the Open Science policy is the adoption of the FAIR principles for research data management and stewardship (FAIR, 2022). The challenge of making the data FAIR will be discussed in terms of SKA and neutron data later in this article.

It is worth comparing progress toward research data management in both the US and UK. In 2013, John Holdren, then director of the US Office of Science and Technology Policy (OSTP), issued a memorandum requiring all major Federal Funding Agencies develop plans to make available the direct results of federally funded scientific research for the public, industry, and the scientific community (Holdren, 2013). Such results include peer-reviewed publications and digital data. The memorandum defined digital data as:

“*the digital recorded factual material commonly accepted in the scientific community as necessary to validate research findings including data sets used to support scholarly publications, but does not include laboratory notebooks, preliminary analyses, drafts of scientific papers, plans for future research, peer review reports, communications with colleagues, or physical objects, such as laboratory specimens.”*

This was one of the earliest attempts to define what “digital data” should be included for research publications. This did not include all the raw observational data taken by experimentalists but only the subset that was relevant to the research publication.

The major US research Funding Agencies have now set up open access repositories and this represents a significant advance toward open access for US research publications. However, the OSTP has recently issued a memorandum^2^ recommending that federal agencies:

“*Update their public access policies as soon as possible, and no later than December 31st, 2025, to make publications and their supporting data resulting from federally funded research publicly accessible without an embargo on their free and public release.”*

Since the US published 17% of peer-reviewed Scientific and Engineering articles in journals and conferences in 2018 (NSF, 2020), this OSTP directive is likely to have a major impact on both publishers and researchers globally.

In the UK, the UK Research and Innovation funding agency has also recently issued its official open access policy for peer-reviewed research articles as well as for monographs, book chapters, and edited collections (UKRI, 2022a). However, in both the US and the UK, the policy on data is less clear. In the UK, each of the seven research councils has its own data management policy although all research proposals are now required to include a Data Management Plan (DCC, 2022). UKRI does now require a “Data access statement” with research publications giving information as to how the supporting data for the reported research may be accessed (UKRI, 2022b). At a global level, currently 5.8% of all articles in Scopus have a link to a dataset (Scopus, 2020).

At the UK's National Laboratories, figures from their research publications repository ePubs^3^ and the Web of Science Data Citation Index indicate a clear upward trend in the number of articles authored or co-authored by staff that now include Data Access Statements (see Table 1). The figures were obtained by downloading article data from ePubs which have Digital Object Identifiers (DOIs), and then running the DOIs through Web of Science.

**Table 1:** Research articles in the ePubs repository indexed in the Web of Science that have DOIs (20^th^ April 2022).

**Year of publication**	**Number of records returned in WoS**	**Articles with related data (WoS)**
2019	1,328	42
2020	1,375	64
2021	1,234	92

## FAIR data: Schema.org, Bioschemas, and W3C DCAT

The FAIR Data Principles are intended as a guide to making data Findable, Accessible, Interoperable and Reusable (FAIR, 2022). The *machine actionability* of the metadata associated with the data is an important aspect of FAIR data, referring to the ability of machines – and not just humans – to understand and manipulate the data. Until fairly recently, search engines had to scrape the Web using keywords and make a best guess about the actual page content. Even after applying natural language processing techniques to a page, there could still be significant ambiguity as to its content. To overcome this, the search engine companies collaborated in the development of the Schema.org vocabulary, a standard vocabulary of generic terms^4^. This allows web developers to provide a high-level overview of the content of the page by embedding machine processable markup within the page source. For example, for a web page about the movie Casablanca, the markup can specify that it was a Movie type (https://schema.org/Movie) and not a city in North Africa. The markup could also provide additional properties such as the title of the movie using (https://schema.org/name) and the names of the actors (https://schema.org/actor). This embedded markup is then used to create the knowledge graphs of the search engines. While the Schema.org vocabulary is not a World Wide Web Consortium (W3C) Recommendation, there is a W3C community group that oversees the development of the Schema.org^5^.

In this paper, the focus is on describing the improvements in the Findability of scientific datasets through the embedding of machine interpretable markup within Web pages as well as improving dataset Interoperability from the use of a common vocabulary of terms. Note that the Schema.org markup only provides a high-level description of what the dataset contains but not all the scientific details required to fully interpret the data. However, the key advance of such web vocabularies is that the markup can be accessed using standard Web protocols and the data can be made available in the JSON-LD format. Users can therefore access data without needing to understand site specific APIs. It is important to stress that since the entry barrier for the data provider is very low, the long-tail of scientific datasets and not just major data providers in a particular field can easily be made accessible.

Although the main focus of Schema.org is to support general Web search, Google offers a dedicated search portal, Google Dataset Search, for collections of data^6^. The Dataset Search Portal allows the user to use a standard keyword search interface to search for collections of data. The content of the Google Dataset Search Portal is obtained from individual web pages using the Schema.org Dataset type. Details of the datasets found can then be shown as well as links to related research articles and the data download location. The corpus of the Google Dataset Search Portal has grown from 500 thousand records in 2016 to 28 million in 2020 (Benjelloun et al., 2020). The portal now contains datasets from a wide range of fields with the Social Sciences (about 26%) and Geosciences (about 19%) dominating.

The Findability of datasets can thus be enhanced by embedding Dataset markup within the homepage of the dataset^7^. When this markup is crawled by Google, the dataset will be added to their internal knowledge graph and become discoverable through their dedicated search portal for datasets as well as for their main search results. However, this search is based on keyword summaries of the dataset and, for example, can only find datasets about diseases in general and not for any specific disease. The Bioschemas initiative is an attempt by the life sciences community to extend the Schema.org vocabulary with specific types relevant to life sciences to enable a slightly deeper inspection of the contents of the resources (Gray et al., in press)^8^. This provides a representation of the key characteristics of the resource and enables an initial level of **I**nteroperability. Nevertheless, to obtain the full set of features of the data, the dataset needs to be retrieved from its original source in the detailed representation format in which it is published. Thus, the Bioschemas proposals do not replace any of the many existing domain ontologies.

The development of the Bioschemas types has followed the philosophy of the Schema.org vocabulary. Instead of trying to accurately capture all the underlying biology, the developed types only aim to capture the characteristics that are most widely used when searching for a concept. In addition, the Bioschemas initiative has developed community agreed usage profiles over the Schema.org types. These profiles identify the core set of properties (typically about 10) to describe a resource of a specific type from the sometimes 100s of available properties for the type. Web page developers simply follow the profile rather than needing to pick and choose which properties to use. The profiles also increase the consistency of the markup available to consuming applications meaning that the data is more viable for Reuse.

Since its inception in 2015, Bioschemas has developed 23 types for describing life sciences concepts, of which 6 have now been included into Schema.org. The community has also defined 37 profiles over these and existing Schema.org types with the goal of making them more accessible to life sciences resource providers. By limiting the number of properties, the process of developing markup for a site is simplified and allows developers to focus more on modeling their own data (Gray et al., in press).

In summary, a key benefit of building on the Schema.org vocabulary is that it is a globally agreed model for representing data. The Schema.org approach, while not addressing important domain specific details, makes the data usable beyond the immediate community of interest, and due to the low deployment effort needed, this approach can be usefully applied to the long-tail of small datasets. Data marked up using Schema.org can be consumed both by community specific registries and wider cross-domain registries, thus dramatically increasing the reach of the data.

Google Dataset Search also supports datasets described using the Data Catalog (DCAT) vocabulary, which has been a World Wide Web Consortium (W3C) recommendation since 2014 (DCAT, 2022). Thus, any webpages describing datasets using the DCAT vocabulary are also crawled by Google and the datasets are displayed *via* their dedicated data search engine.

DCAT's initial version (DCAT v1.0 from 2014) targeted governmental data repositories but later versions have extended the DCAT vocabulary to include terminology that is more relevant for research data. For instance, DCAT v2.0 (a W3C Recommendation since 2020) now covers all the required terms for data citation, guidelines on identifiers, licensing and access rights, dataset quality information, properties to describe temporal and spatial resolutions of datasets. In addition, DCAT has been made more generic by supporting the cataloging and description of any Web Resource, and in particular, the description and cataloging of DataServices. The latest version, currently undergoing the recommendation process, includes treatment of versioning, dataset series and multiple other improvements.

DCAT is being used extensively in many data portals around the world (e.g., Europeana, Zenodo, governmental data catalogs), and the vocabulary itself has been extended *via* Application Profiles to add restrictions and terminologies required in specific domains (e.g., for statistical and geographical data).

## Data management at the ISIS neutron source

Large-scale scientific facilities such as synchrotrons, neutron sources and lasers produce massive amounts of data, which are continuing to grow as their technology improves. Efficiently managing their data throughout its lifecycle is a fundamental activity to enable the science they produce. These management activities range from defining and maintaining a data policy, enabling data discovery and access, up to data archiving and preservation. The new Neutron Beam Line Center at the planned MPR facility is planning the development of a whole new software stack for neutron science (Marais, 2022). With the South Africa draft policy on Open Science this must now require the creation of FAIR datasets. The present Safari-1 team are collaborating with the ISIS Neutron Source at the Rutherford Appleton Laboratory (ISIS, 2022) and with other European neutron sources in the EU BrightnESS^2^ project to bring together the neutron ecosystem for sustainable science^9^. It therefore seems useful to briefly describe how teams in ISIS and in the Scientific Computing Department at the laboratory develop, maintain and run the data management services for the ISIS data catalog (SCD, 2022).

The data management processes implement the ISIS open data policy (ISIS Data Policy, 2022), which is reviewed yearly and updated to reflect any changes in the practices and/or wider open data policy constraints. The data associated with experiments is given a Digital Object Identifier (DOI), which enables data citation^10^. Datasets landing pages are made available *via* a user-friendly interface. The DataGateway interface^11^ provides access to the ISIS data catalog and enables browsing, searching and retrieving embargoed data to its owners and all of the open data to anyone in the world.

The backend system is composed of modular components of the ICAT ecosystem (ICAT Project, 2022). ICAT is an open-source collaborative project across multiple scientific facilities that need this type of data management. The ICAT project revolves around a metadata catalog component, a data retrieval module, a user-friendly interface and other components enabling searches, DOI creation, and so forth. Data may be stored and archived on disks or on tape, depending on the volume and access patterns. In the case of ISIS, the data is kept on disks. Other facilities that are part of the ICAT collaboration are the Diamond Light Source, the European Synchrotron Radiation Facility (ESRF), the ILL Neutron source, the Helmholtz-Zentrum Berlin für Materialien und Energie (HZB), the ALBA Synchrotron Light Source, and the Scientific Computing Department at RAL. These facilities share code, best practices and experiences *via* the ICAT collaboration, as well as collaborating *via* other European projects such as the ExPANDS (2022) and PaNOSC (2022) projects.

In addition to including the Datacite required metadata, the ISIS dataset landing pages have been marked up using the Schema.org mark-up vocabulary, according to Google's guidelines on structured data. The open datasets from the ISIS Neutron source are therefore available using Google's Dataset Search tool. Furthermore, in the ExPANDS project, the ICAT collaboration is planning extensions to the Schema.org vocabulary to better support FAIR data, in a similar fashion to the extensions proposed by the Bioschemas community described above. For photon and neutron datasets, these proposed changes involve adopting the PaNET ontology (NCBO BioPortal, 2022). This provides a taxonomy and thesaurus of photon and neutron (PaN) experimental techniques, based mainly on accelerator-based light sources and neutron facilities. The ontology defines specific techniques in terms of more general technique classes and provides synonyms and references. The goal of using this ontology is to enhance the FAIRness of photon and neutron data catalog services.

The proposed Neutron Beam Line Center at the MPR will make use of similar technologies to those described above to ensure that experimental data generated at the MPR is compliant with the South Africa's draft Open Science policy.

## Thoughts for the future

To support the SKA Science Data Processor and an SKA Regional Center in Africa will require the assembly of an interdisciplinary team consisting of research librarians, data scientists and research software engineers who are collectively skilled in complementary aspects of research data management. While the need for data scientists and software engineers is self-evident it is worth discussing the changing role of librarians in a time when almost all content is born digital.

The LIBER Consortium of research libraries (LIBER Europe, 2022) sees a role for libraries in four key areas of research infrastructure:

- Shared services and Cloud services.
- Semantic interoperability, open and linked data.
- Data stewardship.
- Disciplinary partnerships.

By developing such skills in research data management, libraries can continue to play a central role in supporting first-class research not only at universities but also at national and international facilities. However, this move will require librarians who are qualified to act as domain-specific data stewards to work collaboratively with research scientists, as well as with software engineers and data engineers, to support the emerging research data infrastructure. Research librarians can then play a central role in supporting scientists in coping with the requirements of FAIR data with actionable metadata and semantics.

In terms of the required skills in research software engineering in Africa, there are a number of activities already underway including:

- Research Software & Systems Engineers of Africa^12^.
- Research Software Engineering (RSE) Group at Stellenbosch University, South Africa^13^.

These communities are also liaising with the UK Society of Research Software Engineering^14^ and the UK Software Sustainability Institute (Software Sustainability Institute, 2022). The latter has contributed to the provision of training activities including the software, data and library carpentries^15^.

The ambitious objective of the European Open Science Cloud (EOSC) (European Commission, 2020) is to provide researchers, innovators, companies and citizens with a federated and open multi-disciplinary environment where they can publish, find and reuse data, tools and services for research, innovation and educational purposes. In addition, EOSC has launched the FAIR4S project^16^ to help organizations identify the capabilities and skills required to implement the FAIR principles. EOSC ultimately aims to develop a “Web of FAIR Data and services” for science in Europe upon which a wide range of value-added services can be built.

An initiative to create a Global Open Science Cloud is being promoted by the CODATA organization (Global Open Science Cloud, 2022). CODATA is also supporting the development of a strategy and vision for an African Open Science Platform (AOSP) (African Open Science Platform, 2022):

“*The Platform's mission is to put African scientists at the cutting edge of contemporary, data-intensive science as a fundamental resource for a modern society”*.

The SKA-Mid project will provide a unique opportunity to make the AOSP vision a reality.

## Data availability statement

No new data were generated or analysed during this study.

## Author contributions

The author confirms being the sole contributor of this work and has approved it for publication.

## Funding

This work was partially supported by the Facilities Funding from Science and Technology Facilities Council (STFC) of UKRI, and Wave 1 of the UKRI Strategic Priorities Fund under the EPSRC grant EP/T001569/1, particularly the ‘AI for Science' theme within that grant, by the Alan Turing Institute.

## Conflict of interest

The author declares that the research was conducted in the absence of any commercial or financial relationships that could be construed as a potential conflict of interest.

## Publisher's note

All claims expressed in this article are solely those of the authors and do not necessarily represent those of their affiliated organizations, or those of the publisher, the editors and the reviewers. Any product that may be evaluated in this article, or claim that may be made by its manufacturer, is not guaranteed or endorsed by the publisher.

## Footnotes

^1^https://www.skatelescope.org/wp-content/uploads/2021/03/22380_SKA_Est-Delivery-Plan_DIGITAL_v3.pdf p. 62–63 (accessed July 2022).

^2^https://www.whitehouse.gov/wp-content/uploads/2022/08/08-2022-OSTP-Public-Access-Memo.pdf (accessed October 2022).

^3^The ePubs repository: https://epubs.stfc.ac.uk/ (accessed June 2022).

^4^https://schema.org/ (accessed June 2022).

^5^https://www.w3c.org/community/schemaorg (accessed June 2022).

^6^https://datasetsearch.research.google.com/ (accessed April 2022).

^7^https://schema.org/Dataset (accessed June 2022).

^8^https://bioschemas.org (accessed June 2022).

^9^https://brightness.esss.se/ (accessed October 2022).

^10^https://www.isis.stfc.ac.uk/Pages/Digital-Object-Identifiers-(DOIs)-for-ISIS-Data.aspx (accessed June 2022).

^11^https://data.isis.stfc.ac.uk/ (accessed June 2022).

^12^https://rsse.africa/ (accessed June 2022).

^13^https://rse-at-sun.github.io/RSE-at-SUN/ (accessed June 2022).

^14^https://society-rse.org/ (accessed June 2022).

^15^https://carpentries.org/ (accessed June 2022).

^16^https://www.eoscpilot.eu/sites/default/files/fair4s_eoscpilot_skills_framework.pdf (accessed June 2022).
